# Altered expression of a putative progenitor cell marker DCAMKL1 in the rat gastric mucosa in regeneration, metaplasia and dysplasia

**DOI:** 10.1186/1471-230X-10-65

**Published:** 2010-06-18

**Authors:** Miho Kikuchi, Hiroshi Nagata, Norihito Watanabe, Hiromitsu Watanabe, Masayuki Tatemichi, Toshifumi Hibi

**Affiliations:** 1 Department of Internal Medicine, Keio University School of Medicine, Tokyo, Japan; 2 Department of Internal Medicine, Keiyu Hospital, Kanagawa, Japan; 3 Department of Internal Medicine, Tokai University School of Medicine, Isehara, Japan; 4 Department of Experimental Oncology, Research Institute for Radiation Biology and Medicine, Hiroshima University School of Medicine, Hiroshima, Japan; 5 Department of Hygiene and Preventive Medicine, Showa University School of Medicine, Tokyo, Japan

## Abstract

**Background:**

Doublecortin and calcium/calmodulin-dependent protein kinase-like-1 (DCAMKL1) is a candidate marker for progenitor cells in the gastrointestinal mucosa. Lineage cells in the gastric mucosa are derived from progenitor cells, but this process can be altered after injury. Therefore, we explored DCAMKL1 expression under pathological conditions.

**Methods:**

An immunohistochemical analysis was performed in rat stomach with acute superficial injury, chronic ulcer, intestinal metaplasia and dysplasia.

**Results:**

DCAMKL1 was exclusively expressed in immature quiescent cells in the isthmus of normal fundic glands, where putative progenitor cells are thought to reside. DCAMKL1-positive cells and proliferating cells shed into the lumen after superficial injury and re-appeared during the regenerative process, mainly in the superficial mucosa. In the marginal mucosa around the active ulcer, parietal and chief cells diminished, foveolar hyperplasia was evident, and trefoil factor family 2 (TFF2)/spasmolytic polypeptide-expressing metaplasia (SPEM) emerged at the gland base. DCAMKL1 cells re-emerged in the deep mucosa juxtaposed with SPEM and proliferating cells. In the healing ulcer, the TFF2 cell population expanded and seemed to redifferentiate to chief cells, while proliferating cells and DCAMKL1 cells appeared above and below the TFF2 cells to promote healing. SPEM appeared and PCNA cells increased in the intestinalized mucosa, and DCAMKL1 was expressed in the proximity of the PCNA cells in the deep mucosa. DCAMKL1, PCNA and TFF2 were expressed in different dysplastic cells lining dilated glands near SPEM.

**Conclusion:**

The ultrastructural appearance of DCAMKL1-positive cells and the expression patterns of DCAMKL1 in normal and pathological states indicate that the cells belong to a progenitor cell population. DCAMKL1 expression is closely associated with TFF2/SPEM cells after injury. DCAMKL1 cells repopulate close to proliferating, hyperplastic, metaplastic and dysplastic cells, and the progenitor zone shifts according to the pathological circumstances.

## Background

The mouse and human gastric unit shows monoclonal conversion, indicating the presence of multipotent stem cells[1,2]. Electron microscopic autoradiography in mouse have implied that granule-free cells in the isthmus act as multipotent stem cells[3]. The differentiation and migration processes of cell lineages can be altered by injury. The progenitor cell zone in the isthmus is easily damaged by intraluminal ethanol, non-steroidal anti-inflammatory drugs or *Helicobacter pylori *(*H. pylori*). Chronic inflammation of the stomach can lead to atrophy and specialized cell loss as tangible effects of tissue-specific progenitor cell injury or loss. However, the behavior of progenitor cells after acute or chronic mucosal damage and the mechanism of restoration of these cells during mucosal regeneration are not well understood.

The progenitor cell population is important in maintenance and regeneration of the gastric epithelium, but long-lived progenitor cells are at risk of accumulating mutations that lead to cancer[4]. Neoplasia can follow cellular metaplasia due to chronic inflammation and repair. However, precise analysis of the role and alteration of progenitor cells in the sequence of gastritis-metaplasia-dysplasia-cancer has not been performed, mainly due to the lack of discrete progenitor cell markers in the stomach.

Musashi-1, a marker of progenitor cells in the mouse small intestine, is not expressed in putative progenitor cells, but is found in parietal cells in the rat fundic isthmus[5]. The villin-1 promoter/enhancer fragment is a marker of possible gastric progenitor cells in the isthmus of the pyloric glands[6]. A lineage study indicated that the intestinal progenitor cell marker Lgr5 is expressed at the base of prospective fundic and pyloric glands in the neonatal stomach, whereas expression in adult was predominantly restricted to the base of the pyloric glands[7]. Thus, there are no definite markers for progenitors in adult fundic glands.

DCAMKL1 is one of the products of Gene Ontogeny-enriched transcripts found in comparison with mouse gastric and small intestinal progenitor datasets[8]. Immunohistochemical analysis using a DCAMKL1 antibody revealed single cell staining in intestinal crypt sections at or near position 4 and in gastric isthmus cells[8]. After the first report, specific localization of DCAMKL1-expressing cells in a stem cell niche was shown in the small intestine of mice[9,10] and in the colon of mice and humans[11,12], whereas DCAMKL1 was coexpressed with Musashi-1 in parietal cells in the stomach of mice[13].

The first aim of this study was to determine whether DCAMKL1 is a marker for progenitor cells in the rat stomach. The second aim was to elucidate the temporal and spatial patterns of appearance of cells specifically expressing DCAMKL1 under several gastric pathological conditions.

## Methods

### Animal Preparation

All animal protocols were approved by the Keio University Animal Research Committee. Male Wistar rats weighing about 200 g were fasted for 24 hours with free access to water. To produce acute superficial injury in the fundic mucosa, absolute ethanol (1 ml) was instilled into the stomach by gastric intubation. To produce chronic deep ulcers, 20% acetic acid (50 μl) was injected into the fundic submucosa of the anterior wall using a microsyringe. The rats were euthanized 3 days and 1, 2 and 3 weeks after the acetic acid injection.

The method for inducing intestinal metaplasia in the rat gastric mucosa has been described elsewhere[14,15]. Briefly, rats received two X-ray doses of 10 Gy each and were euthanized 6 months after irradiation. The method for inducing dysplasia in the rat gastric mucosa has also been described elsewhere[14]. Briefly, 50 μg/ml of N-methyl-N'-nitro-N-nitrosoguanidine (MNNG) (Aldrich Chemical Co., Milwaukee, WI) was given to rats *ad libitum *for 4 months.

### Antibodies

Rabbit anti-DCAMKL1 immunoglobulin (Ig)G (Abcam, Cambridge, UK; final dilution 1:100), mouse anti-proliferating cell nuclear antigen (PCNA) IgG (DAKO, Carpinteria, CA; ready to use), rabbit anti-PCNA IgG (Abcam; 1:200), mouse anti-H^+^/K^+^-adenosine triphosphatase (ATPase) α subunit IgG (Research Diagnostics Inc. Flanders, NJ; 1:200), sheep anti-pepsinogen II IgG (United States Biological, Swampscott, MA; 1:100), mouse anti-MUC6 IgM (Kanto Kagaku, Tokyo; 1:100), mouse anti-MUC5AC IgG (Abcam; 1:100), mouse anti-TFF2 IgM (Abcam; 1:200), guinea pig anti-histidine decarboxylase (HDC) IgG (ARP, Belmont, MA; 1:100), goat anti-ghrelin IgG (Santa Cruz Biotechnology, Santa Cruz, CA; 1:100), and mouse anti-somatostatin IgG (Biomeda, Foster City, CA; 1:25) were used as primary antibodies.

### Histological Analysis

The stomach tissue was fixed in 10% neutral-buffered formalin overnight, and then embedded in paraffin and sectioned (4 μm). The sections were stained with hematoxylin and eosin (H&E) using standard techniques. Periodic acid-Schiff (PAS)-Alcian Blue staining was performed to detect mucous cell lineages.

For immunohistochemical analysis, paraffin-embedded sections were deparaffinized and pretreated with an appropriate retrieval procedure for each antigen. Sections were incubated in 0.3% H_2_O_2 _in methanol for 10 minutes to inactivate endogenous peroxidase and then washed with phosphate-buffered saline (PBS) containing 0.1% Tween 20 (PBST). After incubation with blocking solution (Block Ace, Dainippon Seiyaku, Tokyo, Japan) for 10 minutes, the sections were incubated with a primary antibody for 1 hour at room temperature. Thereafter, each step was followed by washing 3 times with PBST for 3 minutes. The sections were incubated with HP-conjugated IgG or IgM for 40 minutes. The labeled cells were colored brown with 3,3'-diaminobenzidine hydrochloride (DAB) using a DAB reagent set (DAKO), and then counterstained with Mayer's hematoxylin.

For double-color immunostaining, an indirect immunoalkaline phosphatase method was used following the indirect immunoperoxidase procedure described above. After reaction with DAB, the sections were incubated with a second primary antibody for 1 hour, followed by incubation with ALP-conjugated IgG or IgM for 40 minutes. The labeled cells were stained blue with an ALP substrate kit III (Vector Blue, Vector Laboratories, Burlingame, CA).

For antigen retrieval of pepsinogen II and MUC6, proteinase K (DAKO) was applied topically to the deparaffinized sections for 6 minutes. For antigen retrieval of PCNA (mouse IgG), the sections were heated in distilled water in an autoclave for 10 minutes. For antigen retrieval of MUC5AC, the sections were heated in citrate buffer in an autoclave for 10 minutes. No antigen retrieval procedure was used for DCAMKL1, H^+^/K^+^-ATPase, PCNA (rabbit IgG), TFF2, HDC, ghrelin or somatostatin staining.

### Scoring of DCAMKL1 Cells and PCNA Cells

Sections that underwent double-color immunostaining using DCAMKL1 and PCNA were analyzed to determine the number of immunostained cells. Sections were used from 5 rats, and 10 well-oriented gastric units were analyzed in each section.

### Electron Microscopy

Pre-embedding immunoperoxidase electron microscopy was carried out as follows. Small pieces of fresh specimens from the gastric corpus of untreated rats were fixed in 4% paraformaldehyde for 24 hours, followed by fixation in 0.1% glutaraldehyde and 4% paraformaldehyde for 1 hour. Cryostat sections (6 μm) were prepared and incubated with anti-DCAMKL1 antibody for 48 hours, followed by incubation with HP-conjugated anti-rabbit IgG for 24 hours. The sections were then fixed with 0.5% glutaraldehyde for 5 minutes, reacted with DAB, post-fixed with 2% osmium tetroxide, dehydrated through a graded ethanol series, and embedded in epoxy resin. Ultrathin sections were cut with an ultramicrotome and stained in uranyl acetate solution and lead citrate solution. The specimens were examined using a transmission electron microscope (JEM-1200EX, JEOL, Tokyo, Japan).

## Results

### Normal Fundic Gland

**Figure 1:**
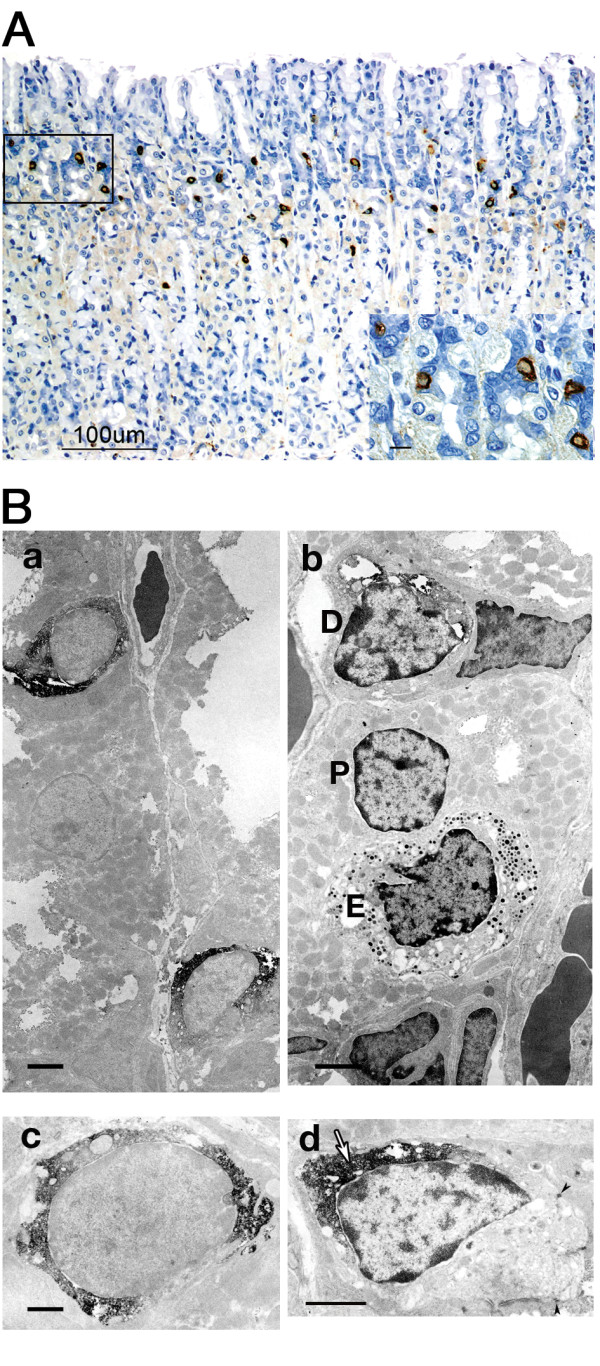
**Distribution and ultrastructure of DCAMKL1-expressing cells in normal rat stomach**. (A) A light micrograph, showing location of DCAMKL1 cells in the fundic mucosa. Scale: 100 μm. The inset shows a magnified view of the outlined area in A. Scale: 10 μm. (B) Transmission electron micrographs. (a, c) Ultrathin sections without counterstaining. (b, d) Ultrathin sections with counterstaining. (a) DCAMKL1 cells in the fundic gland. Scale: 2 μm, magnification ×5600. (b) The DCAMKL1 cell (D) was smaller than the parietal cell (P), which was rich in mitochondria, and lacked the secretory granules seen in the endocrine cell (E). Scale: 2 μm, magnification ×7000. (c) DCAMKL1 staining was predominantly cytoplasmic. Scale: 1 μm, magnification ×14400. (d) Arrowheads indicate desmosomes. The white arrow shows DCAMKL1 immunoreactivity. Scale: 2 μm, magnification ×8400.

DCAMKL1-expressing cells were distributed in the upper third of normal fundic glands, in a region referred to as the isthmus (Figure 1A). In electron microscopy, DCAMKL1-cells were also found in the isthmus (Figure 1B, a). The DCAMKL1 cell was smaller than the parietal cell, which was rich in mitochondria, and lacked the secretory granules seen in the endocrine cell (Figure 1B, b). DCAMKL1 immunoreactivity was found diffusely in the cytoplasm, giving a high nucleocytoplasmic ratio (Figure 1B, c). DCAMKL1 cells were present in epithelial cell linings, since the cells had desmosomes in the junction with adjacent epithelial cells (Figure 1B, d). The DCAMKL1 cells had an immature appearance with few mitochondria, vesicles or secretory granules (Figure 1B, c and 1B, d).

**Figure 2:**
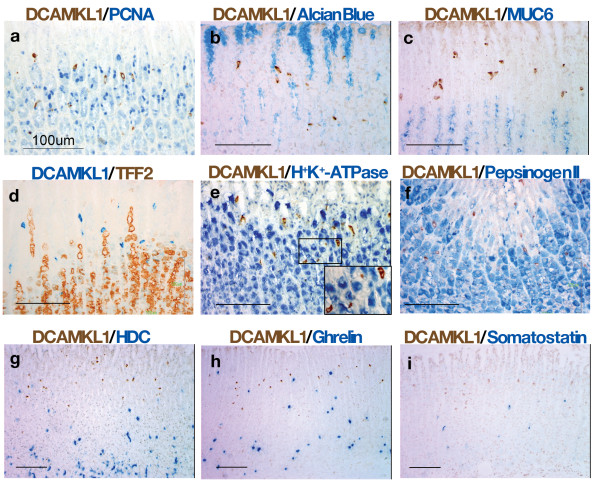
**Double-color immunostaining showing localization of DCAMKL1-expressing cells and epithelial cell lineages comprising proliferating cells with PCNA-labeled nuclei (a), Alcian Blue-stained foveolar cells (b), MUC6-stained mucous neck cells (c), TFF2-stained mucous neck cells (d), H^+^/K^+^-ATPase-stained parietal cells (e), pepsinogen II-stained chief cells (f), HDC-stained enterochromaffin-like cells (g), ghrelin-stained A-like cells (h), and somatostatin-stained D cells (i)**. Scales: 100 μm. The inset in (e) shows a magnified view of the outlined area. Note that the DCAMKL1 cells were distinct from the parietal cells.

We next compared the distribution of DCAMKL1 cells with those of known epithelial cell lineages. DCAMKL1 cells were intermingled with proliferating cells labeled by PCNA in the isthmus (Figure 2a), but no DCAMKL1 cells contained PCNA. DCAMKL1 cells resided below foveolar cells (stained with Alcian Blue, Figure 2b) and above mucous neck cells (stained with MUC6 and TFF2, Figure 2c, d). Parietal cells and chief cells in the isthmus were adjacent to DCAMKL1 cells, but DCAMKL1 cells did not coexpress H+/K+-ATPase or pepsinogen (Figure 2e, f). DCAMKL1 cells were also discrete from endocrine cell lineages. The DCAMKL1 cell population was distant from enterochromaffin-like cells, which were principally distributed in the fundic base (Figure 2g). A majority of A-like cells resided in the fundic base with some distributed in the isthmus but distinct from DCAMKL1 cells (Figure 2h), and D cells clearly differed from DCAMKL1 cells (Figure 2i).

### Acute Superficial Mucosal Injury and Rapid Renewal

**Figure 3:**
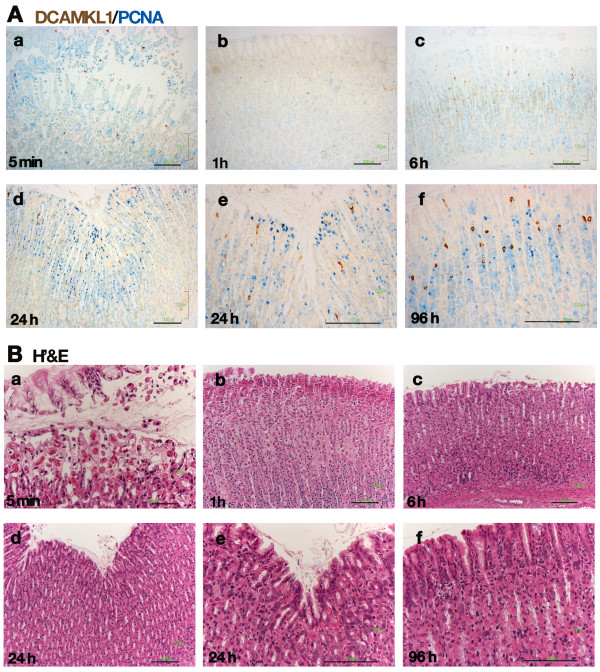
**Immunohistochemical analysis of superficial mucosal injury after ethanol treatment**. (A) Double-color immunostaining for DCAMKL1 cells and PCNA cells. Gastric sections taken at 5 minutes (a), 1 hour (b), 6 hours (c), 24 hours (d, e) and 96 hours (f) after ethanol treatment. (B) H&E-stained sections, showing the time course of gastric mucosal damage and repair after ethanol administration. (a-f) Serial sections of the respective sections in A. Scales: 100 μm.

Histological analysis of the process of mucosal injury and repair after ethanol administration using H&E staining and double-color DCAMKL1 and PCNA immunostaining is shown in Figure 3. Immediately after ethanol treatment, DCAMKL1 cells and PCNA cells detached from the gland and shed into the lumen with foveolar cells (Figure 3Aa and 3Ba). DCAMKL1 cells and PCNA cells had almost disappeared in the damaged mucosa 1 hour after ethanol treatment (Figure 3Ab and 3Bb). DCAMKL1 cells then reappeared after 6 hours, and some were present near the mucosal surface (Figure 3Ac and 3Bc). PCNA cells increased at 24 hours after ethanol (Figure 3Ad and 3Bd), while several DCAMKL1 cells and PCNA cells were present at the mucosal surface (Figure 3Ae and 3Be). The distribution of DCAMKL1 cells and PCNA cells had the morphologic appearance of untreated fundic mucosa after 96 h (Figure 3Af and 3Bf).

**Figure 4:**
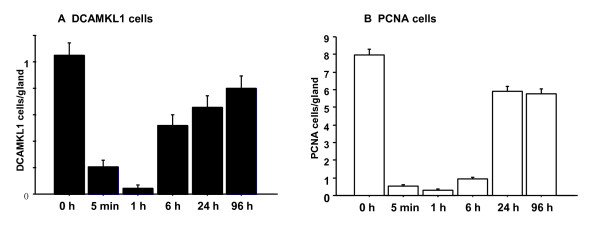
**Time courses of the numbers of DCAMKL1 cells (A) and PCNA cells (B) in a gland after ethanol administration**. Each bar represents the mean ± SE of results from 5 rats. The Y-axis shows the number of cells per gland and the X-axis shows the time after ethanol administration.

Time courses of changes in the numbers of DCAMKL1 and PCNA cells are shown in Figure 4. The number of DCAMKL1 cells changed almost concomitantly with that of PCNA cells, but recruitment of DCAMKL1 cells began at 6 hours after ethanol treatment, prior to recruitment of PCNA cells.

### Chronic Ulcer

**Figure 5:**
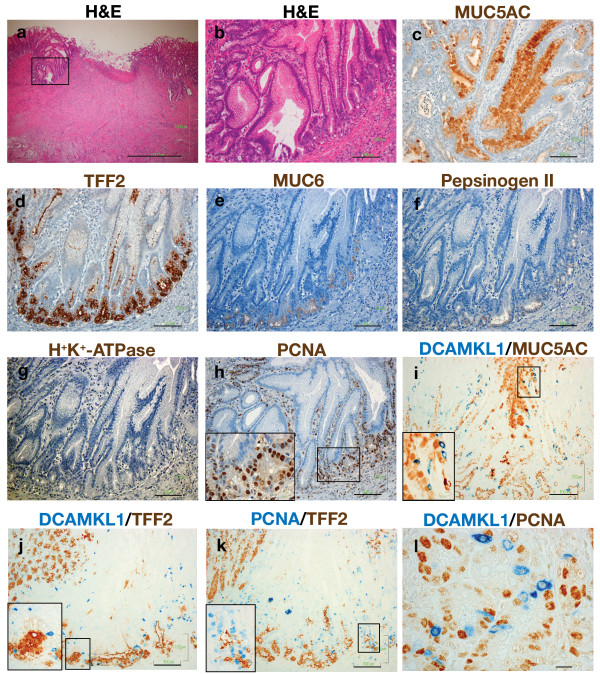
**Patterns of epithelial cells in the marginal mucosa of an active ulcer**. (a) An H&E-stained section taken at 1 week after acetic acid treatment, showing a crater of granulation tissue and marginal tissue surrounding the crater. Scale: 1000 μm. (b) A magnified view of the ulcer margin mucosa outlined in (a). Scale: 100 μm. (c-h) Serial sections of (b) stained with MUC5AC (c), TFF2 (d), MUC6 (e), pepsinogen II (f), H^+^/K^+^-ATPase (g), and PCNA (h). (i-k) Double-color immunostaining for DCAMKL1 with MUC5AC (i), DCAMKL1 with TFF2 (j), and PCNA with TFF2 (k). Scales: 100 μm. (l) Double-color immunostaining for DCAMKL1 with PCNA. Scale: 10 μm. The inset in (h-k) shows a magnified view of the outlined area.

Deep ulcers involving whole mucosal layers and penetrating the muscularis mucosa were produced 3 days after treatment with acetic acid. Most ulcers healed after 2 weeks of treatment and some recurred after 3 weeks. A histopathological analysis of mucosal regeneration was performed using tissues taken at 1 to 3 weeks after treatment. Cystically dilated glands were prominent in the regenerative mucosa of the ulcer margin around the crater of an active ulcer (Figure 5a, b). These glands were lined with cells expressing MUC5AC, a marker of foveolar cells (Figure 5c). In addition, TFF2, a marker of mucous neck cells in untreated rats, displayed intense staining at the base of regenerative fundic glands (Figure 5d), similar to that for TFF2 staining of deep antral gland cells and consistent with the emergence of a SPEM cell phenotype[16,17]. In contrast, expression of MUC6, another marker of mucous neck cells in untreated rats, deteriorated in the regenerative mucosa and only weak MUC6 staining was observed in cells at the gland base (Figure 5e). Chief cells also diminished in the ulcer margin and cells weakly stained with pepsinogen were visualized only at the gland base (Figure 5f). Thus, there was overlap in expression of TFF2, MUC6 and pepsinogen in cells at the base (Figure 5, d-f). Parietal cells were absent in the tissue of the marginal mucosa of the active ulcer (Figure 5g). PCNA cells increased in the glands and mesenchyma of the ulcer margin and a marked increase in these cells was noted at the gland base (Figure 5h).

We then explored DCAMKL1 expression in the marginal mucosa of the active ulcer. Dispersed DCAMKL1-expressing cells were present close to MUC5AC cell linings (Figure 5i) and juxtaposed with SPEM cells (Figure 5j). PCNA cells were also distributed in the vicinity of SPEM cells and some SPEM cells coexpressed PCNA (Figure 5k), implying that the SPEM lineage is multiplying and proliferative. DCAMKL1 cells were intermingled with PCNA cells at the base of the gland, but did not coexpress PCNA (Figure 5l). This indicates that DCAMKL1 cells are maintained in a quiescent state. The progenitor zone of PCNA cells and DCAMKL1 cells was displaced from the isthmus in the normal gland to the base in the margin of the active ulcer.

**Figure 6:**
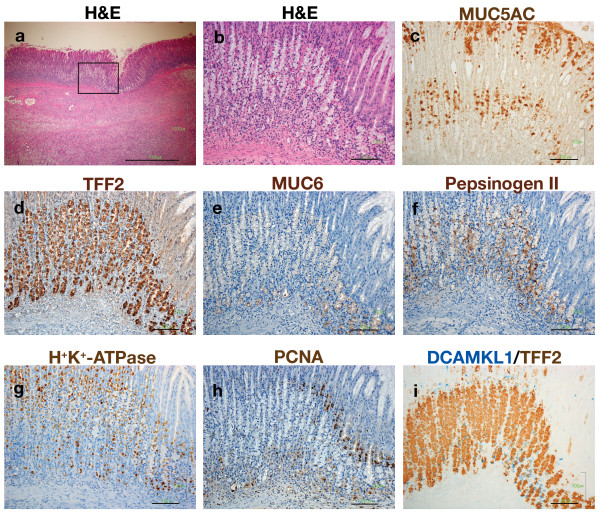
**Patterns of epithelial cells and DCAMKL1 cells in the regenerative mucosa of a healing ulcer**. (a) An H&E-stained section taken at 2 weeks after acetic acid treatment, showing the regenerative tissue of the healing ulcer. Scale: 1000 μm. (b) A magnified view of the regenerative mucosa outlined in (a). A gradient of epithelial regeneration from the ulcer edge (right side) to the regenerated mucosa distant from the ulcer (left side) was identified. Scale: 100 μm. (c-h) Serial sections of (b), stained with MUC5AC (c), TFF2 (d), MUC6 (e), pepsinogen II (f), H^+^/K^+^-ATPase (g), and PCNA (h). (i) Double-color immunostaining for DCAMKL1 and TFF2 in a serial section of (b).

In the healing stage, the ulcer size reduced and epithelial cells were restored (Figure 6a). In the healing ulcer, a gradient of epithelial regeneration was present from the ulcer edge to the regenerated glands distant from the ulcer (Figure 6b). In the regenerative mucosa of the healing ulcer, mucous-like cells markedly increased. These cells were localized at the base in the ulcer margin and expanded to the necks of glands as healing proceeded. MUC5AC cells decreased in the neck and was present mainly in the foveolae (Figure 6c), similar to the location in the normal fundic mucosa. The expanding mucous-like cells in the healing ulcer were partly stained by MUC5AC, but predominantly stained with TFF2 (Figure 6d). MUC6 was expressed in cells at the gland base but not in those in the neck (Figure 6e), whereas pepsinogen was expressed in cells in the neck (Figure 6f). Parietal cells, which had vanished in the active ulcer, reappeared above and below the TFF2 cell population in the mucosa of the healing ulcer (Figure 6g). In the normal mucosa, parietal cells are derived from progenitor cells in the isthmus, mature therein, and then take several days to migrate down to the base[18], whereas in the healing ulcer, parietal cells repopulated the neck and base of the gland simultaneously. PCNA was expressed strongly just above TFF2 cells near the ulcer, indicating enhanced amplification of the epithelial cells in this region (Figure 6h). Some PCNA cells were also distributed below the TFF2 cells. DCAMKL1 cells reappeared above and juxtaposed with the lower half of the TFF2 cell population (Figure 6i). This dual distribution profile of the progenitor zone may contribute to promotion of rapid and efficient mucosal regeneration.

### Intestinal Metaplasia and Dysplasia

**Figure 7:**
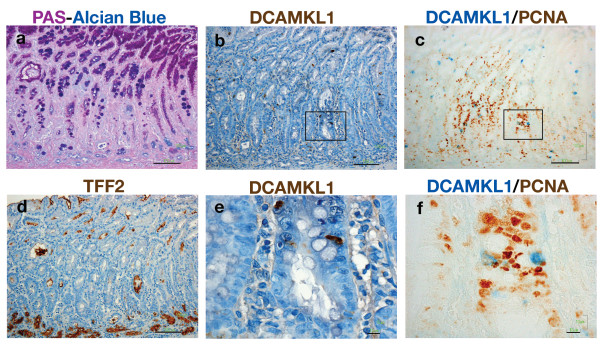
**DCAMKL1 expression in the intestinalized mucosa**. (a) PAS-Alcian Blue staining showing the mucosa with intestinal metaplasia containing goblet cells. Scale: 100 μm. (b-d) Serial sections of (a), stained with DCAMKL1 (b), DCAMKL1 and PCNA (c), and TFF2 (d). (e, f) Magnified views of the areas outlined in (b) and (c), respectively. Scales: 10 μm.

In irradiated rats, intestinal metaplasia containing goblet cells identified by PAS-Alcian Blue staining formed in the fundic gland (Figure 7a). Cells expressing DCAMKL1 were found below foveolar cells in the luminal compartment of the mucosa, as well as in the deep mucosa (Figure 7b, c). PCNA cells increased markedly in the intestinalized mucosa and DCAMKL1 cells in the luminal compartment were distal to the PCNA cells (Figure 7c). TFF2-expressing cells, consistent with SPEM, emerged at the base of the intestinalized mucosa (Figure 7d). PCNA cells were found proximal to DCAMKL1 cells deep in the intestinalized epithelial lining, with a similar distribution pattern to that in the small intestinal crypt (Figure 7e, f).

**Figure 8:**
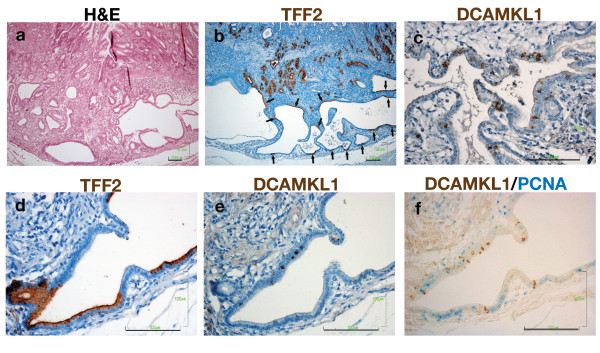
**DCAMKL1 expression in the dilated glands with dysplasia**. (a) An H&E-stained section, showing cystic dilation of glands. (b) Expansion of SPEM. Arrows indicate segmental expression of TFF2 in the dilated glands. (c) Expression of DCAMKL1 in the dilated gland. (d-f) Serial sections of the dilated gland for TFF2 (d), DCAMKL1 (e) and DCAMKL1 with PCNA (f). Scales: 100 μm.

In rats treated with MNNG, cystic dilation of glands with dysplasia was elicited in the mucosa and submucosa (Figure 8a). SPEM evolved and expanded near the dilated glands and segmental expression of TFF2 was found in cells lining the glands (Figure 8b). DCAMKL1 was sparsely expressed in these glands (Figure 8c). TFF2 and DCAMKL1 were expressed in different cells in the dilated glands (Figure 8d, 8e), and PCNA was also expressed in cells other than DCAMKL1 cells, indicating the proliferative nature of the glands (Figure 8f).

## Discussion

The study showed that DCAMKL1-expressing cells are exclusively present in the isthmus of the rat fundic gland, in which multipotent progenitor cells are thought to reside[1,3]. We demonstrated that DCAMKL1 cells are discrete from differentiated epithelial cells, including endocrine cell lineages. Furthermore, immunoelectron microscopy showed that DCAMKL1 was expressed in immature epithelial cells with few organelles, which correspond to the granule-free cells previously proposed as presumptive progenitor cells in the gastric isthmus[3]. DCAMKL1 is coexpressed with Musashi-1 in parietal cells in the mouse stomach[13], whereas this study demonstrated that DCAMKL1 cells were distinct from parietal cells, which express Musashi-1 in the rat stomach[5].

**Figure 9:**
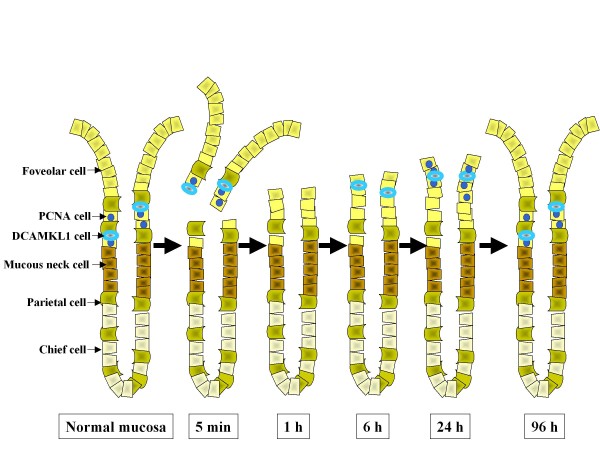
**Sequential changes in cellular loss and recovery of the gastric gland after superficial mucosal injury with ethanol**.

Sequential changes in cellular loss and recovery of the gastric gland after superficial mucosal injury with ethanol are summarized in Figure 9. The time courses of changes in the numbers of DCAMKL1 and PCNA cells 6 to 96 hours after ethanol treatment in rat are consistent with those in mouse[13]. In addition, we analyzed earlier changes in these cell types. DCAMKL1 cells and PCNA cells desquamated with foveolar cells immediately after ethanol treatment. A denuded mucosal surface after ethanol exposure is re-epithelialized in 1 to 2 hours by rapidly migrating foveolar cells from nearby uninjured epithelium[19]. Since this early restitution is not based on cellular proliferation but on migration, mobilization of progenitor cells is not required. After a latent period of approximately 8 hours, a burst of proliferative activity occurred until 24 hours to restore the foveolae to their original length[20]. This time course of epithelial proliferation after ethanol exposure is similar to that observed in the present study.

A recent report has shown that PCNA-positive proliferating cells contain prefoveolar cells[21]. The increased number of proliferating cells are probably derived from progenitor cells, but the process of progenitor cell repopulation after injury is obscure. In this study, DCAMKL1 cells were recruited prior to restoration of proliferating cells, and the number and distribution of DCAMKL1 cells changed almost concomitantly with those of proliferating cells after 24 hours. This finding implies that DCAMKL1-expressing cells are progenitor cells that give rise to proliferating cells. Several DCAMKL1 cells and PCNA cells were present at the mucosal surface during the early regenerative period. Transient displacement of the progenitor zone toward the surface may help to facilitate rapid and preferential restoration of foveolar cells, which is the lineage that is most damaged and lost in superficial mucosal injury. Since the recruited DCAMKL1 cells reappeared within a day after the indigenous cells were lost, the repopulating DCAMKL1 cells in the injured mucosa may migrate from non-injured glands or recruit from progeny of a multipotent stem cell lineage that undergoes continuous expansion or extinction in the niche[9,22].

**Figure 10:**
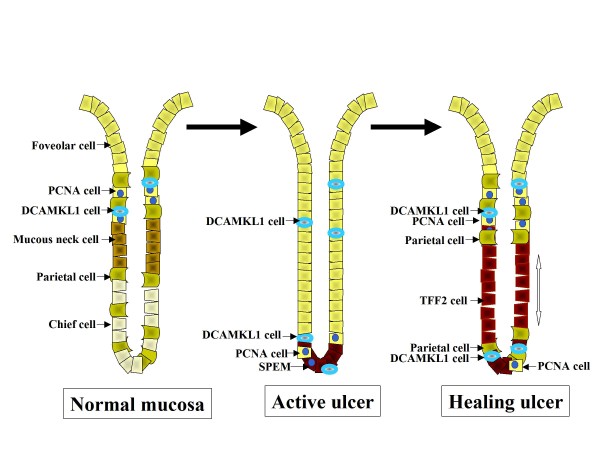
**Alterations of mucosal cell lineages in the margin of active and healing ulcers**.

The processes of mucosal reconstruction and progenitor cell repopulation in a chronic deep ulcer differed clearly from those in acute superficial injury (Figure 10). Differentiated cell lineages are maintained after acute superficial injury, whereas orderly differentiation of mucosal cell lineages in the active ulcer was perturbed and inherent cell lineages were replaced by newly developed mucous cell lineages. Parietal and chief cells were lost, foveolar hyperplasia was evident, and SPEM emerged in the regenerating epithelium surrounding the active ulcer. These findings mimic pathological changes in various experimental models induced by acute or chronic oxyntic atrophy[16,17,23,24]. In the ulcer margin, MUC6 and pepsinogen seem to be coexpressed with TFF2 at the gland base. This finding supports the hypothesis that chief cells transdifferentiate to SPEM[17,25], and that the process of redifferentiation from mucous neck cells to chief cells is modified in an active ulcer. An alternative explanation, which is more likely based on the present results, is that SPEM is derived from progenitor cells and redifferentiates to chief cells. The finding that SPEM is associated with increased proliferation supports this proposal[16,24].

Chronic mucosal ulceration in the gastrointestinal tract initiates a healing process from the mucosal base at the ulcer edge, and can induce novel cell lineages corresponding to SPEM[26]. These appear to be derived from multipotent stem cells in the crypt of the small intestine and colon, whereas SPEM and proliferating cells at the base of the margin of the gastric ulcer is distant from the normal progenitor zone in the isthmus. The chronic ulcer model in this study developed several weeks after treatment, which makes migration of bone marrow-derived stem cells unlikely because engraftment of these cells as gastric epithelial cells occurs in mice after sustained *H. felis *infection over more than 1 year, but not in mice with a gastric ulcer induced by acetic acid[27]. The presence of a second progenitor population, cryptic progenitor cells in the stomach, has been predicted for many years[16,24], but has yet to be identified. In this study, putative progenitor DCAMKL1 cells were found in the vicinity of two mucous cell lineages, foveolar cells and SPEM cells, in hyperplasia at the ulcer margin. DCAMKL1 cells juxtaposed with SPEM are compatible with the cryptic progenitor cells. The intestinal progenitor cell marker Lgr5 is present at the base of glands and not in the isthmus[7], and that a progenitor cell population increased markedly during inflammation[6]. We hypothesize that DCAMKL1-expressing cells at the base of the gastric gland are the second-line progenitor population, which is masked under physiological conditions. These dormant progenitors may be activated by inflammatory cytokines during ulcer formation, and may play a pivotal role in initiating the healing process.

DCAMKL1 cells and PCNA cells were present close to TFF2/SPEM cells in the ulcer margin. TFF2 has a healing function in the stomach[28], but the mechanism is not fully understood. The function of TFF2, which is protease resistant and produces mucus/TFF complexes of high viscosity, might be to protect the progenitor zone [29]. During ulcer healing, the TFF2 cell population expanded from the base to the neck of glands. Unlike mucous neck cells in the normal mucosa, TFF2 cells in the healing ulcer coexpressed MUC5AC (but little MUC6) in the neck and also coexpressed pepsinogen. This suggests that SPEM cells redifferentiate to chief cells in the process of ulcer healing.

**Figure 11:**
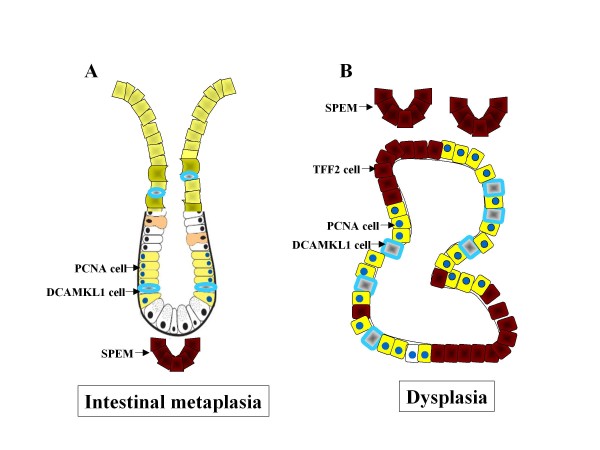
**Alterations of mucosal cell lineages in intestinal metaplasia (A) and dysplasia (B)**.

Alterations of mucosal cell lineages in intestinal metaplasia are illustrated in Figure 11A. Intestinal metaplasia with goblet cells was induced by irradiation in the fundic mucosa, whereas SPEM developed at the base. Many cells in the intestinalized mucosa showed proliferation, and the marked proliferation of these cells is coincident with that in human atrophic gastritis[30]. In the non-treated rat, proliferating cells were present in the proximity of DCAMKL1 cells in the isthmus, whereas in the irradiated rat, the proliferative zone shifted downward and was separate from the DCMKL1 cell population in the luminal compartment. On the other hand, PCNA cells were found in the proximity of DCAMKL1 cells deep in the intestinalized mucosa. The arrangement of DCAMKL1 cells and proliferating cells in the intestinalized mucosa mimics that in the small intestinal crypt. These findings indicate that DCAMKL1 is a common progenitor cell marker in the gastrointestinal tract. It will be of interest to determine how gastric stem cells become reprogrammed to enter a differentiation program characteristic of the small intestine.

Alterations of cell lineages in dysplasia are illustrated in Figure 11B. Cystic dilation of glands was elicited and the morphological appearance was consistent with gastritis cystic profunda as a precursor to dysplasia and neoplasia, which develops in *Helicobacter*-infected animals and humans[23,31]. The cystic regions invaded the submucosa and were accompanied by SPEM, which also expanded to the submucosa from the base of the mucosa. It is of note that several studies have suggested that SPEM is a precursor of gastric cancer[23,27,32]. DCAMKL1 and TFF2 were expressed in dilated glands in this study. Expression of DCAMKL1 has been reported in mouse adenoma[9] and human colorectal cancer[12], and thus DCAMKL1 and TFF2 are putative markers for gastric carcinogenesis. A recent report showed that DCAMKL1 is a positive regulator of tumorigenesis in the colon[12], but the function of DCAMKL1 in the gastrointestinal tract is largely unknown.

## Conclusions

The ultrastructural appearance of DCAMKL1 cells and the expression patterns of DCAMKL1 in normal and pathological states indicate that the cells belong to a progenitor cell population. DCAMKL1 shows different expression profiles in ulcer healing, intestinal metaplasia and dysplasia, while DCAMKL1 expression is closely associated with TFF2/SPEM cells.

DCAMKL1 is expressed close to proliferating, hyperplastic, metaplastic and dysplastic cells, and the progenitor zone shifts according to the pathological circumstances.

## Competing interests

The authors declare that they have no competing interests.

## Authors' contributions

MK carried out immunostaining and drafted the manuscript. HN designed the study and revised the manuscript. NW carried out electron microscopy. HW and MT carried out animal experiments and prepared sections. TH supervised the study. All authors read and approved the final manuscript.

## Pre-publication history

The pre-publication history for this paper can be accessed here:

http://www.biomedcentral.com/1471-230X/10/65/prepub
